# Supplementary material for: The genome sequence of the Sprawler moth, Asteroscopus sphinx Hufnagel, 1766
Source: Wellcome Open Res. 2024 Oct 11;9:505. Originally published 2024 Sep 3. [Version 2] doi: 10.12688/wellcomeopenres.22920.2 (PMC11589413; doi:10.12688/wellcomeopenres.22920.2)
Supplement: Peer review history

# Reviewer report: Reviewer response for version 2

**DOI:** 10.21956/wellcomeopenres.25588.r110986

**Published:** 2024-11-30

**Authors:** Bryan Brunet

**Affiliations:** 1 Ottawa Research and Development Centre, Ontario, Canada

The article entitled "The genome sequence of the Sprawler moth, Asteroscopus sphinx Hufnagel, 1766" by author J. Hammond presents a chromosome-scale genome assembly as well as mitogenome assembly for the sprawler moth. With a few minor exceptions, the methods used are either sufficiently described or referenced in cited literature. The exceptions being the methods used in the determination of the Z and B chromosomes, as well as the cobiont and contaminants pipeline. This information is necessary to support the author's deductions and allow for replication of workflows that are currently not publicly available. Otherwise, the genome assembly appears to be of high quality and should provide an important scientific contribution, albeit some additional background information on this taxon and its life history would help to provide context and justify the importance of assembling its genome. There are also several minor revisions requested, listed below:

 1) Use consistent units - Gb and Gbp are both used in the same paragraph of the genome sequence report.

 2) Please explain how the Z and B chromosomes were identified as such.

 3) Indicate whether reads or assembled contigs were used as input into mitohifi. If contigs, were scaffolds of mitochondrial origin identified using Blobtools?

 4) Describe the cobionts and contaminants pipeline and/or provide a url to its code repository. It is insufficient to say it is in prep without providing sufficient detail to allow for replication of the steps taken.

Are sufficient details of methods and materials provided to allow replication by others?

Partly

Is the rationale for creating the dataset(s) clearly described?

Partly

Are the datasets clearly presented in a useable and accessible format?

Yes

Are the protocols appropriate and is the work technically sound?

Yes

Reviewer Expertise:

aphid systematics & taxonomy, genomics

I confirm that I have read this submission and believe that I have an appropriate level of expertise to confirm that it is of an acceptable scientific standard.

# Reviewer report: Reviewer response for version 2

**DOI:** 10.21956/wellcomeopenres.25588.r110983

**Published:** 2024-11-26

**Authors:** Panagiotis Ioannidis

**Affiliations:** 1 Foundation for Research & Technology - Hellas, Crete, Greece

This manuscript by Hammond describes the sequencing and assembly of the genome of the lepidopteran Asteroscopus sphinx. All the metrics suggest that it is a fairly complete genome assembly that is almost ready to be used for downstream analyses.

 I believe that a bit more background on the B chromosomes (section "Genome sequence report") would be very helpful for the non-specialist reader, in order to understand what B chromosomes are, and why they are important.

 The author mentions that the genome will be annotated using already-generated RNAseq data (section "Data availability"). My understanding is that this annotation will probably take a while, so I've been wondering why the author didn't use Ensembl Rapid Release to quickly generate a gene set until a "proper" one (i.e. using RNAseq data) is generated. I understand that not using RNAseq data results in a gene set of sub-optimal quality, but at least you will have a gene set to work with (and you can always run BUSCO to make sure it doesn't miss a lot of conserved genes).

Are sufficient details of methods and materials provided to allow replication by others?

Yes

Is the rationale for creating the dataset(s) clearly described?

Yes

Are the datasets clearly presented in a useable and accessible format?

Yes

Are the protocols appropriate and is the work technically sound?

Yes

Reviewer Expertise:

Insect genomics, bioinformatics

I confirm that I have read this submission and believe that I have an appropriate level of expertise to confirm that it is of an acceptable scientific standard.

# Reviewer report: Reviewer response for version 2

**DOI:** 10.21956/wellcomeopenres.25588.r110990

**Published:** 2024-11-25

**Authors:** Sarah Inwood

**Affiliations:** 1 University of Otago, Dunedin, Otago, New Zealand

- The authors sequenced and assembled a chromosomal scale genome assembly for the Sprawler moth *Asteroscopus sphinx*. This assembly used HiFi long reads and Hi-C data and technically sound methodology, resulting in a high-quality nuclear genome assembly, alongside a mitochondrial genome assembly. 
- The background section is relatively brief compared to most Darwin Tree of Life genome assembly data notes. ​This section would greatly benefit from incorporating more details about the biology of *A. sphinx*, such as native range information, ecological role and life cycle & life history which could broaden the appeal of the manuscript. If this information is not available (thus explaining the brief background), the lack of research on the species of interest could be stated by the authors to further highlight the potential value of an assembled genome.

As a minor note, the software tools table is missing a version number for MultiQC.

Are sufficient details of methods and materials provided to allow replication by others?

Yes

Is the rationale for creating the dataset(s) clearly described?

Yes

Are the datasets clearly presented in a useable and accessible format?

Yes

Are the protocols appropriate and is the work technically sound?

Yes

Reviewer Expertise:

Genomics, host-parasite interactions, parasitoid wasps, insects, viruses, biocontrol

I confirm that I have read this submission and believe that I have an appropriate level of expertise to confirm that it is of an acceptable scientific standard.

# Reviewer report: Reviewer response for version 1

**DOI:** 10.21956/wellcomeopenres.25237.r100422

**Published:** 2024-10-04

**Authors:** Pritha Dey

**Affiliations:** 1 National Centre for Biological Sciences-TIFR, Bangalore, India

I found the article to be technically very sound, and the detailed methods will allow future research to replicate them. The article is also vey important and timely, in the era where new genome resources are expanding possibilities for developing insect model systems.

 However, I felt in the background section, the species taxonomy, life history and ecology is missing. This is crucial information which along with the genome resources would be critical for further research.

 In the sample acquisition sections under 'Methods', it is mentioned that an adult female was acquired. Whereas, in the background it is written that the genome was assembled from a male. Could the authors kindly clarify?

Are sufficient details of methods and materials provided to allow replication by others?

Yes

Is the rationale for creating the dataset(s) clearly described?

Yes

Are the datasets clearly presented in a useable and accessible format?

Yes

Are the protocols appropriate and is the work technically sound?

Yes

Reviewer Expertise:

A moth-biologist, interested in diversity, distribution and evolutionary ecology of moths.

I confirm that I have read this submission and believe that I have an appropriate level of expertise to confirm that it is of an acceptable scientific standard.
